# Role of Escin in breast cancer therapy: potential mechanism for inducing ferroptosis and synergistic antitumor activity with cisplatin

**DOI:** 10.1007/s10495-023-01849-x

**Published:** 2023-05-06

**Authors:** Chenyuan Li, Ziqi He, Feng Yao, Shichong Liao, Kai Sun, Shengrong Sun, Zhiyu Li, Zhong Wang

**Affiliations:** 1 Department of Breast & Thyroid Surgery, Renmin Hospital of Wuhan University, Wuhan, Hubei People’s Republic of China; 2 Department of Urology, Renmin Hospital of Wuhan University, Wuhan, 430060 Hubei People’s Republic of China

**Keywords:** Breast cancer, Escin, Ferroptosis, G6PD, Cisplatin

## Abstract

Breast cancer (BC) has threatened women worldwide for a long time, and novel treatments are needed. Ferroptosis is a new form of regulated cell death that is a potential therapeutic target for BC. In this study, we identified Escin, a traditional Chinese medicine, as a possible supplement for existing chemotherapy strategies. Escin inhibited BC cell growth in vitro and in vivo, and ferroptosis is probable to be the main cause for Escin-induced cell death. Mechanistically, Escin significantly downregulated the protein level of GPX4, while overexpression of GPX4 could reverse the ferroptosis triggered by Escin. Further study revealed that Escin could promote G6PD ubiquitination and degradation, thus inhibiting the expression of GPX4 and contributing to the ferroptosis. Moreover, proteasome inhibitor MG132 or G6PD overexpression could partially reverse Escin-induced ferroptosis, when G6PD knockdown aggravated that. In vivo study also supported that downregulation of G6PD exacerbated tumor growth inhibition by Escin. Finally, our data showed that cell apoptosis was dramatically elevated by Escin combined with cisplatin in BC cells. Taken together, these results suggest that Escin inhibits tumor growth in vivo and in vitro via regulating the ferroptosis mediated by G6PD/GPX4 axis. Our findings provide a promising therapeutic strategy for BC.

## Introduction

Breast cancer (BC) is the most common malignant tumor and the leading cause of death among women globally [1]. BC accounts for over 25% of new cancer cases in females worldwide and 6.9% of cancer deaths [2]. The current treatments for BC include chemotherapy, targeted therapy, endocrine therapy and other therapies [3]. However, traditional chemotherapeutic drugs cannot always relieve tumor growth and dissemination [4]. Novel treatments remain to be further explored.

Ferroptosis is a new programmed death mode featuring the iron-dependent accumulation of lipid reactive oxygen species (ROS) [5]. Morphologically, ferroptosis involves shrinkage of mitochondria with reduced mitochondrial cristae, condensed membranes and ruptured outer membranes [6]. Mechanistically, imbalanced intracellular iron donates electrons to oxygen to form ROS, thus sensitizing cells to oxidative damage and ferroptosis [7]. Meanwhile, antioxidant defense serves as a classical repressor of ferroptosis. System χ_c_^−^, composed of SLC7A11 (xCT) and SLC3A2, imports extracellular cystine (*Cys2*) in exchange for intracellular glutamate (*Glu*), which is necessary for sustaining glutathione (GSH) levels [8]. Stable GSH guarantees the activity of glutathione peroxidase (GPX4) [9], which is able to directly reduce phospholipid hydroperoxide to hydroxy-phospholipid [10]. It has been reported that ferroptosis plays a crucial role in cancer cell death in cancers such as diffuse large B-cell lymphomas, renal cell carcinoma and BC [11]. Furthermore, combining many chemotherapeutic drugs, including cytarabine, cisplatin, doxorubicin and temozolomide, with the ferroptosis inducer erastin has a dramatic antitumor effect [12]. Thus, ferroptosis inducers could be potential therapeutics for BC treatment.

Glucose-6-phosphate dehydrogenase (G6PD) is the rate-limiting enzyme of the oxidative branch of the pentose phosphate pathway [13]. Steady cell life activity requires appropriate G6PD activity. Low levels of G6PD activity lead to cell death, while aberrant activation of G6PD results in increased ROS levels and chaotic cell growth and differentiation [14]. In terms of ferroptosis, recent studies have suggested that G6PD serves as a hub gene in the biology of ferroptosis and predicts the poor overall survival of many solid tumors [15–17]. Biochemically, it contributes to reductive lipid biosynthesis and the production of GSH [13]. This process is also reflected in the resistance to ROS and ferroptosis in clear cell renal cell carcinoma (ccRCC) [18]. G6PD inhibition sensitizes ccRCC cells to ferroptosis, while increased G6PD activity in mouse kidney tissues manifests as reduced lipid peroxidation [18]. Additionally, impairment of G6PD activity induces apoptosis in erlotinib-resistant pancreatic cancer cells through reduction of NADPH and augmentation of ROS levels [19].

Escin is a traditional medicine extracted from *Aesculus hippocastanum*. It was initially illustrated to have antiedematous, anti-inflammatory and venotonic properties [20]. This traditional Chinese medicine is now known to show an antineoplastic effect. Many studies have claimed that Escin blocks tumor progression in ovarian cancer, pancreatic cancer, lung cancer, BC and other cancers [21–24]. In our previous study, Escin increased ROS levels and triggered autophagy activation [25]. In addition, Escin inhibits cell viability and colony formation and induces DNA damage, cell cycle arrest and apoptosis in human colorectal cancer [25, 26]. Furthermore, Escin improves the progression-free survival and overall survival of patients with either HCC or colon carcinoma [27]. Moreover, Escin synergizes with 5-fluorouracil (5-FU) in BC and with gemcitabine in pancreatic cancer cells [28, 29]. However, it remains elusive whether Escin triggers ferroptosis via G6PD regulation in BC.

Cisplatin (DDP) is an effective chemotherapeutic drug in various cancers. It is able to crosslink DNA purine bases, thereby interfering with DNA repair process, eventually leading to cell death [30]. In addition, DDP also induces cell apoptosis by forming stable complexes with DNA or proteins to prevent cell reduplication. Furthermore, cisplatin was reported to serve as an immunomodulatory agent in response to immune escape and inhibition [30]. However, DDP resistance and the its toxicity remain the major clinical impediments to the treatment of solid cancers. Great efforts have been made and the newest study demonstrated that microRNAs and siRNAs are promising candidates in DDP resistance. They can sensitize cancer cells to DDP via downregulating survivin or oncogenic pathways [31, 32]. Moreover, drug combination can effectively avoid the adverse impacts of DDP. It was reported that curcumin could reduce ROS levels and inhibit inflammation to relieve DDP-mediated side effects [33].

In this context, we investigated the roles of ferroptosis and G6PD in Escin-induced BC cell death and the underlying mechanism. Furthermore, we explored the synergy between Escin and cisplatin. The findings of this research might be useful in developing chemotherapy strategies for BC patients, and Escin could be a supplement to traditional chemotherapeutic drugs.

## Materials and methods

### Cell culture and drug treatment

Human BC cell lines (MDA-MB-231 and MCF7) were obtained from American Type Culture Collection (Manassas VA, USA), which was authenticated by STR profiling. They were cultured with Dulbecco’s modified Eagle’s medium (Gibco, USA) with 10% fetal bovine serum (AusgeneX, Australia) and 1% penicillin/streptomycin at 37 °C in a 5% CO_2_ atmosphere. Escin was obtained from Shandong Luye Pharma LTD (Shandong, China) and dissolved in 0.9% NaCl. MG132 (HY-13259) and cisplatin (DDP, HY-17394) were purchased from MedChemExpress (Shanghai, China) and prepared according to the manufacturer’s instructions.

### Cell growth assays

A CCK8 assay (Biosharp, BS350B) was performed according to the manufacturer’s instructions. A total of 5000 cells/well were seeded in 96-well plates. Cells were treated and incubated with 10 µl CCK8 reagent at 37 °C for 2 h. The absorbance was measured at 450 nm. A colony formation assay was performed according to our previous study [26]. A total of 200 cells/well were seeded in 6-well plates. Then, 5, 10 and 20 μg/ml Escin were added. Approximately 14 days later, colonies were visible and fixed. Cells were stained with 0.1% crystal violet and photographed.

### qRT‒PCR

Total RNA was extracted with TRIzol reagent (Takara, Japan). Reverse transcription was carried out with a TransScript First-Strand cDNA Synthesis Kit (Takara, Japan). qRT‒PCR was performed with SYBR Green MasterMix (Takara, Japan). The following primer sequences were used: G6PD: forward: 5′ CGAGGCCGTCACCAAGAAC, reverse: 5′ GTAGTGGTCGATGCGGTAGA; β-actin: forward: 5′ CTCCATCCTGGCCTCGCTGT, reverse: 5′ GCTGTCACCTTCACCGTTCC.

### siRNA transfection and viral infection

G6PD siRNA (5′ AAACCCACUCUCUUCAUCAGCUCGU) was used to knock down the expression of G6PD, while a scramble siRNA was used as a control. The siRNAs were obtained from GenePharma Co. (Shanghai, China). Flag-G6PD, Flag-GPX4 and their counterparts were purchased from GeneChem Co. (Shanghai, China). Human sh-G6PD and sh-NC lentiviruses were purchased from GeneChem Co. (Shanghai, China).

### Western blot analysis

Western blotting was performed according to a previous study [26]. Primary antibodies against G6PD (1:1000, Proteintech, 25413-1-AP), GPX4 (1:1000, ABclonal, A1933), xCT (1:1000, Proteintech, 26864-1-AP), LC3 (1:5000, MBL International, M186-3) and β-actin (1:5000, Proteintech, 66009-1-Ig) were used.

### Flow cytometry

Apoptosis was detected by an Annexin V FITC/PI Apoptosis Kit (APCC101, MULTI SCIENCES, China) according to the manufacturer’s instructions. The resuspended cells were stained with 10 μl PI and 5 μl FITC at room temperature in the dark for 5 min. Then, the samples were analyzed by a FACScan flow cytometer.

ROS levels were detected by an ROS Assay Kit (S0033S, Beyotime, China) according to the manufacturer’s instructions. Cells were seeded in 6-well plates and treated. DCFH-DA was diluted to 10 μM with serum-free DMEM and added to the wells. The cells were stained at 37 °C in the dark for 20 min. Then, the cells were collected and analyzed by a FACScan flow cytometer.

### Measurement of G6PD activity

The activity of G6PD was detected with a G6PD activity assay kit (S0189, Beyotime, China). The collected cells were resuspended in ice G6PD extraction solution and centrifuged at 12,000×*g* for 5 min at 4 °C. The supernatant was added to 96-well plates with the G6PD substrate and color solution. After 10 min of incubation at 37 °C in the dark, the absorbance at 450 nm was measured.

### Measurement of GSH

Intracellular GSH levels were detected by a reduced GSH assay kit (A006-2-1, Nanjing Jiancheng Bioengineering Institute, China). The collected cell suspension was mixed with Solution I and centrifuged at 3500 rpm for 10 min. The supernatant was added to 96-well plates and reacted with the corresponding solution. The absorbance at 420 nm was measured.

### Measurement of lipid peroxidation

Intracellular lipid peroxidation was detected with BODIPY™ 581/591 C11 (D3861, Thermo Fisher). Treated cells were incubated with 2.5 μM C11 at 37 °C for 30 min in the dark. The cells were washed with PBS 3 times and photographed.

### Electron microscopy

The procedure was carried out according to our previous study [34]. In brief, treated cells were fixed with ice-cold 2.5% glutaraldehyde at 4 °C for 1 h. Then, the cells were collected and centrifuged at 800 rpm for 5 min. The supernatant was discarded, and the cells were resuspended gently. The cell suspension was left to stand and sediment naturally. The supernatant was removed, and new fixation liquid was added gently. Images were obtained by an electron microscope.

### Immunofluorescence (IF)

Treated cells were seeded on cover glass in 24-well plates. After washing with PBS 3 times, the cells were fixed and permeabilized. Then, 2% BSA was used to block the cells for 1 h at room temperature. Cells were incubated with primary antibody against G6PD (1:100, Proteintech, 25413-1-AP) overnight at 4 °C. After washing with 0.05% Triton X-100 3 times, the cells were incubated with 488-conjugated donkey anti-rabbit IgG (1:1000, Jackson ImmunoResearch Laboratories) for 30 min at room temperature and then with DAPI incubation for 3 min. Finally, the cells were cover-slipped with mounting medium (Sigma, F4680, USA).

### Immunohistochemistry (IHC)

The procedure and the scoring rules were performed according to our previous study [34]. Antibodies against G6PD (1:100, Proteintech, 25413-1-AP) and GPX4 (1:100, Proteintech, 67763-1-Ig) were used.

### Hoechst staining

Treated cells were fixed and stained with 10 μg/ml Hoechst 33342 (Beyotime, China). Images were observed by fluorescence microscopy.

### Animal experiments

All animal experiments were approved by the Animal Ethics Committee of Wuhan University (No. WDRM20211001). MDA-MB-231 cells stably expressing sh-NC and sh-G6PD were established. Four-week-old female BALB/c nude mice were bred in Animal Experimental Center of Renmin Hospital of Wuhan University. They were injected with corresponding stable cells (2.5 × 10^6^ cells per mouse) into the right iliac fossa. When the tumor grew to approximately 60 mm^3^, 2 mg/kg Escin was intraperitoneally injected with normal saline as the control. Nude mice were euthanized by exposure to CO_2_ when the tumor volume reached to approximately 300 mm^3^ and the nodules were removed for analysis.

### Statistical analysis

All data were analyzed with three independent experiments and are presented as the mean ± SD. P < 0.05 was considered to indicate statistical significance.

## Results

### Escin induced BC cell death

**Fig. 1:**
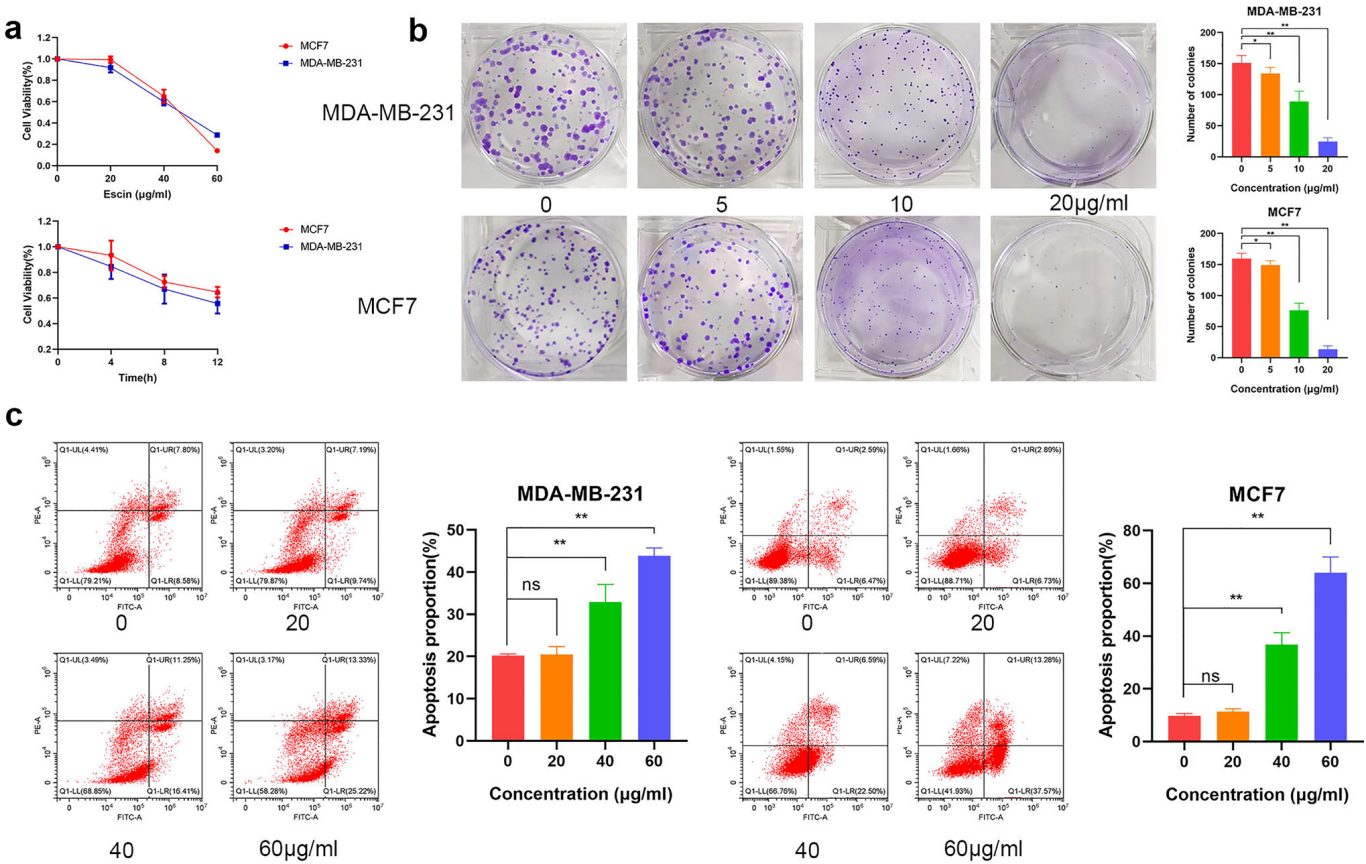
Escin inhibited cell proliferation and induced apoptosis in BC cells. **a** Cells were treated with a series of concentrations of Escin (0, 20, 40 and 60 μg/ml) for 4, 8, and 12 h. Cell viability was measured by CCK8 assay. **b** A total of 200 cells were treated with a series of concentrations of Escin (0, 5, 10 and 20 μg/ml). Colony formation was measured after 14 d of cultivation. **c** Cells were treated with a series of concentrations of Escin for 12 h. Apoptosis was analyzed by flow cytometry. The values are the mean ± SD from 3 independent experiments. ^ns^p > 0.05, *p < 0.05, **p < 0.01 vs. their counterpart

To investigate the toxic effect of Escin on BC cells, a CCK8 assay was used to examine the viability of MDA-MB-231 and MCF7 cells in the presence of Escin according to a concentration gradient and a time gradient. Cell viability obviously decreased in a concentration- and time-dependent manner (Fig. 1a). Similarly, colony formation ability was impaired in a concentration-dependent manner (Fig. 1b). The results of flow cytometry also showed the same trend (Fig. 1c). These results suggested that Escin induced BC cell death.

### Escin induced ferroptosis in BC cells

**Fig. 2:**
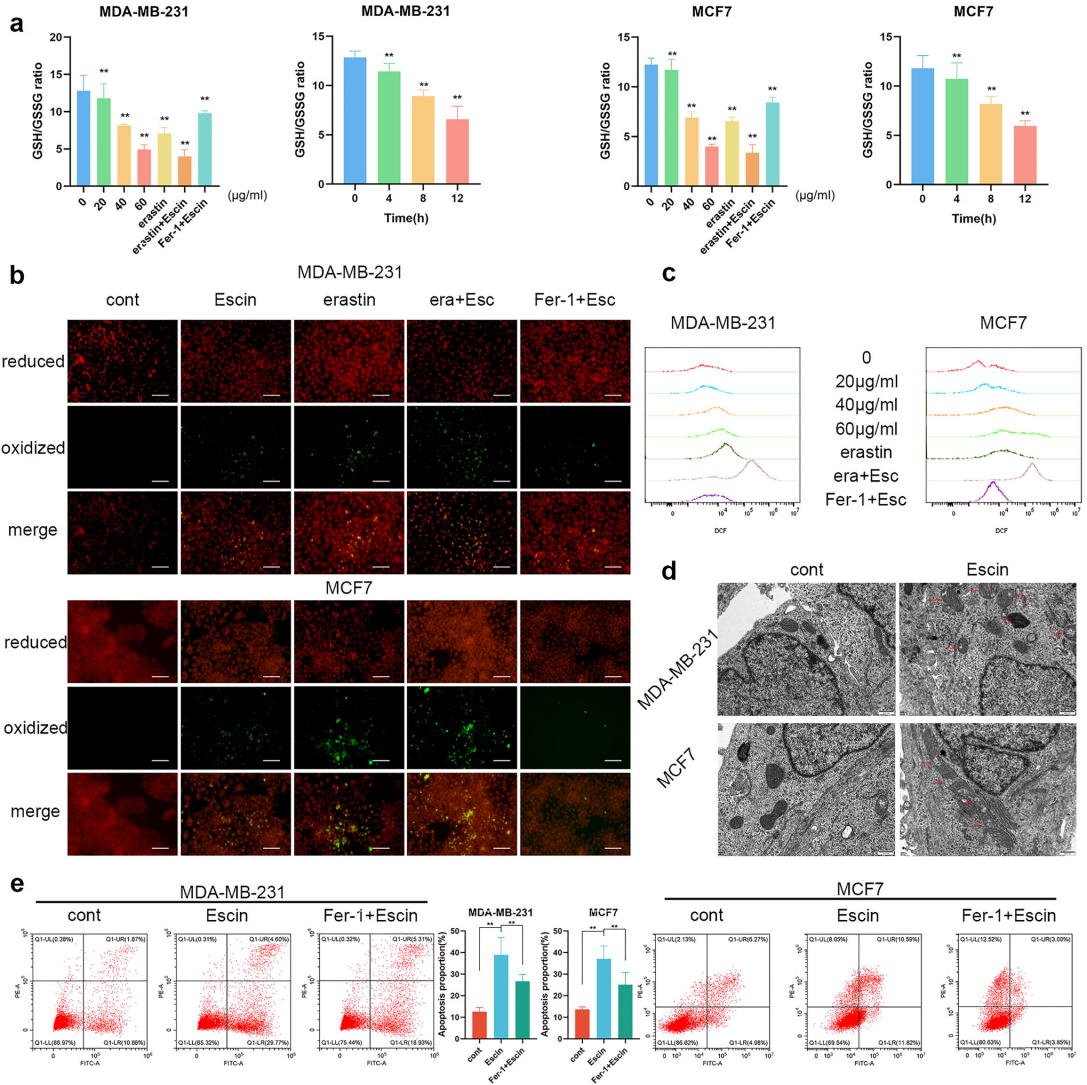
Escin induced ferroptosis in BC cells. **a** GSH/GSSG ratio measurement. As the positive control, cells were treated with 5 μM erastin for 48 h. As the negative control, cells were treated with 1 μM Fer-1 for 2 h. Then, the cells were treated with a series of concentrations of Escin (0, 20, 40 and 60 μg/ml) for 4, 8, and 12 h. **b** Cells were treated as described above. Lipid peroxidation was determined with BODIPY 581/591 C11. **c** ROS levels were determined by flow cytometry. **d** Cells were treated with 40 μg/ml Escin for 12 h. Mitochondria were observed by electron microscopy (8000×, scale = 500 nm). **e** Cells were pretreated with 1 μM Fer-1 for 2 h and then 40 μg/ml Escin for 12 h. Apoptosis was analyzed by flow cytometry

To explain the underlying mechanism of Escin-triggered cell death, we detected a ferroptosis-related index. Cells were treated with 20, 40 and 60 μg/ml Escin for 12 h or with 5 μM erastin for 48 h as the positive control. Cells were pretreated with 1 μM Fer-1 for 2 h as the negative control. The GSH/GSSG ratio decreased in a dose-dependent manner and further decreased when Escin synergized with erastin (Fig. 2a). Similarly, the GSH/GSSG ratio was detected in cells treated with Escin for 4, 8 and 12 h. The ratio was also reduced in a time-dependent manner (Fig. 2a). Additionally, lipid peroxidation was increased by Escin and further increased when combined with erastin (Fig. 2b). The same results were observed with regard to the changes in ROS levels (Fig. 2c). Additionally, the GSH/GSSG ratio, lipid peroxidation and ROS were all reversed by pretreatment with Fer-1 (Fig. 2a–c). Morphologically, cells exposed to Escin exhibited a ruptured mitochondrial outer membrane and reduced mitochondrial cristae (Fig. 2d). Furthermore, pretreatment with the ferroptosis inhibitor Fer-1 alleviated Escin-induced apoptosis (Fig. 2e). These results implied that Escin caused BC cell death via ferroptosis.

### Escin induced ferroptosis via GPX4 inhibition

**Fig. 3:**
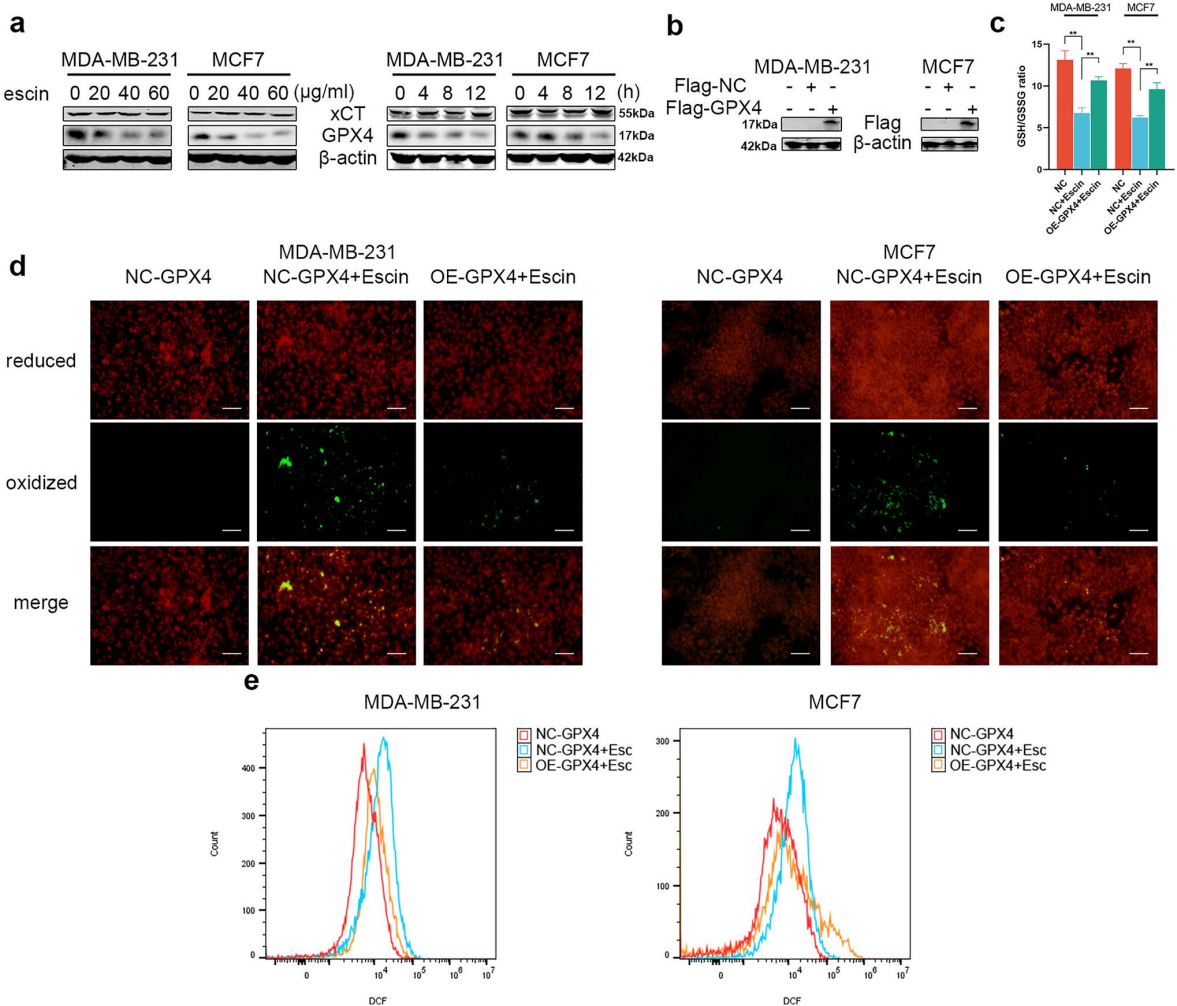
GPX4 was downregulated during Escin-induced ferroptosis. **a** Cells were treated with Escin (0, 20, 40 and 60 μg/ml) for 12 h or 40 μg/ml Escin for 4, 8 and 12 h. The protein levels of xCT and GPX4 were detected by Western blotting. **b** Overexpression efficiency of GPX4 in BC cells. **c**–**e** GSH/GSSG ratio and lipid peroxidation (200×, scale = 100 μm) and ROS measurement after GPX4 overexpression with Escin treatment

To explore how Escin induced ferroptosis, we detected the protein levels of xCT and GPX4. The expression of xCT did not show a significant difference, while the expression of GPX4 dramatically decreased in a concentration- and time-dependent manner (Fig. 3a). Furthermore, BC cells overexpressed GPX4 (Fig. 3b), and the GSH/GSSG ratio, lipid peroxidation level and ROS levels were detected. Overexpression of GPX4 obviously attenuated Escin-induced ferroptosis (Fig. 3c–e). These results suggested that GPX4 was inhibited in Escin-induced ferroptosis.

### Escin promoted the ubiquitination and degradation of G6PD in BC cells

**Fig. 4:**
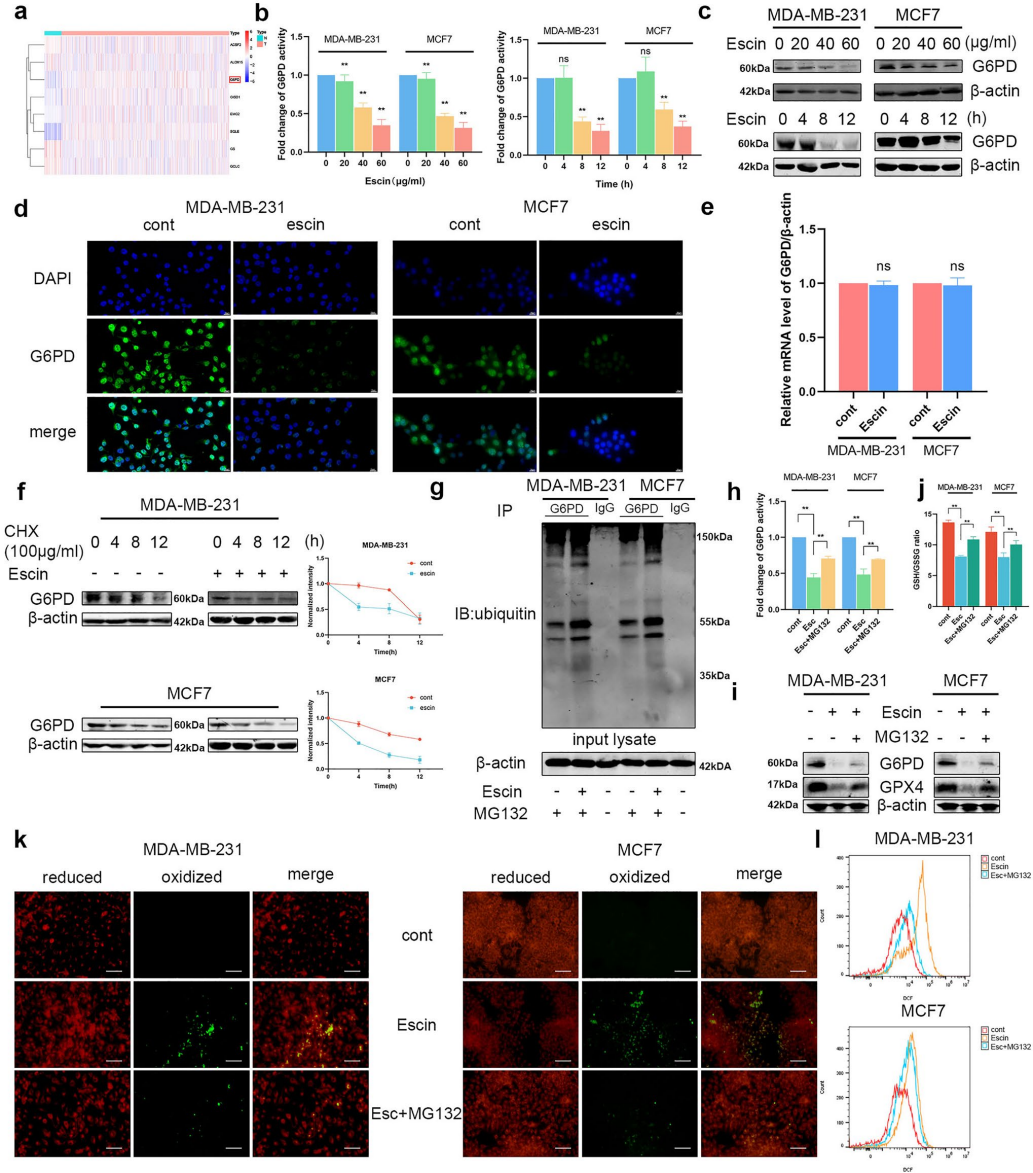
Escin facilitated G6PD ubiquitination and degradation. **a** Heatmap illustrated the ferroptosis-related genes. **b** Cells were treated with Escin (0, 20, 40 and 60 μg/ml) for 12 h or 40 μg/ml Escin for 4, 8 and 12 h. G6PD activity was measured. **c** Cells were treated as described above. The protein level of G6PD was detected by Western blotting. **d** The expression of G6PD was analyzed by IF (400×, scale = 20 μm). **e** The mRNA level of G6PD in Escin-treated BC cells (40 μg/ml, 12 h). **f** Cells were treated with 100 μg/ml CHX for 4, 8 and 12 h in the presence or absence of 40 μg/ml Escin. The protein level of G6PD was detected by Western blotting. **g** BC cells were treated with 20 μM MG132 for 12 h followed by 40 μg/ml Escin for 12 h. The cell lysates were subjected to immunoprecipitation using a G6PD antibody. Then, the coprecipitating endogenous proteins were detected by Western blotting. **h** Cells were pretreated with 20 μM MG132 for 12 h and then treated with 40 μg/ml Escin for 12 h. The activity of G6PD was measured. **i** Cells were treated as described above. The protein levels of G6PD and GPX4 were detected. **j**–**l** Cells were treated as described above. The GSH/GSSG ratio, lipid peroxidation (200×, scale = 100 μm) and ROS levels were measured

Importantly, bioinformatics analysis illustrated that G6PD served as a hub gene of ferroptosis (Fig. 4a), we speculated that Escin induced ferroptosis by inhibiting G6PD activity. Firstly, we detected G6PD activity and protein levels in BC cells treated with Escin. The activity and expression of G6PD both decreased in a dose- and time-dependent manner (Fig. 4b, c). IF staining also suggested that G6PD expression was inhibited by Escin (Fig. 4d). However, mRNA expression was not significantly changed (Fig. 4e). Therefore, we speculated that Escin could increase the degradation of G6PD. We next investigated the stability of G6PD in the presence of Escin and cycloheximide (CHX). Compared with CHX treatment alone, Escin treatment promoted the degradation of G6PD in the presence of CHX (Fig. 4f). Furthermore, coimmunoprecipitation analysis with an anti-G6PD antibody also revealed that Escin increased the ubiquitination of G6PD in the presence of MG132 (Fig. 4g). Moreover, the proteasome inhibitor MG132 reversed the inhibition of G6PD activity by Escin (Fig. 4h), similar to the changes in the protein levels of G6PD and GPX4 (Fig. 4i). Regarding the ferroptosis-related index, the GSH/GSSG ratio, lipid peroxidation and ROS levels were all reversed by MG132 (Fig. 4j–l). In brief, Escin accelerated the ubiquitination of G6PD, thereby inducing ferroptosis.

**Fig. 5:**
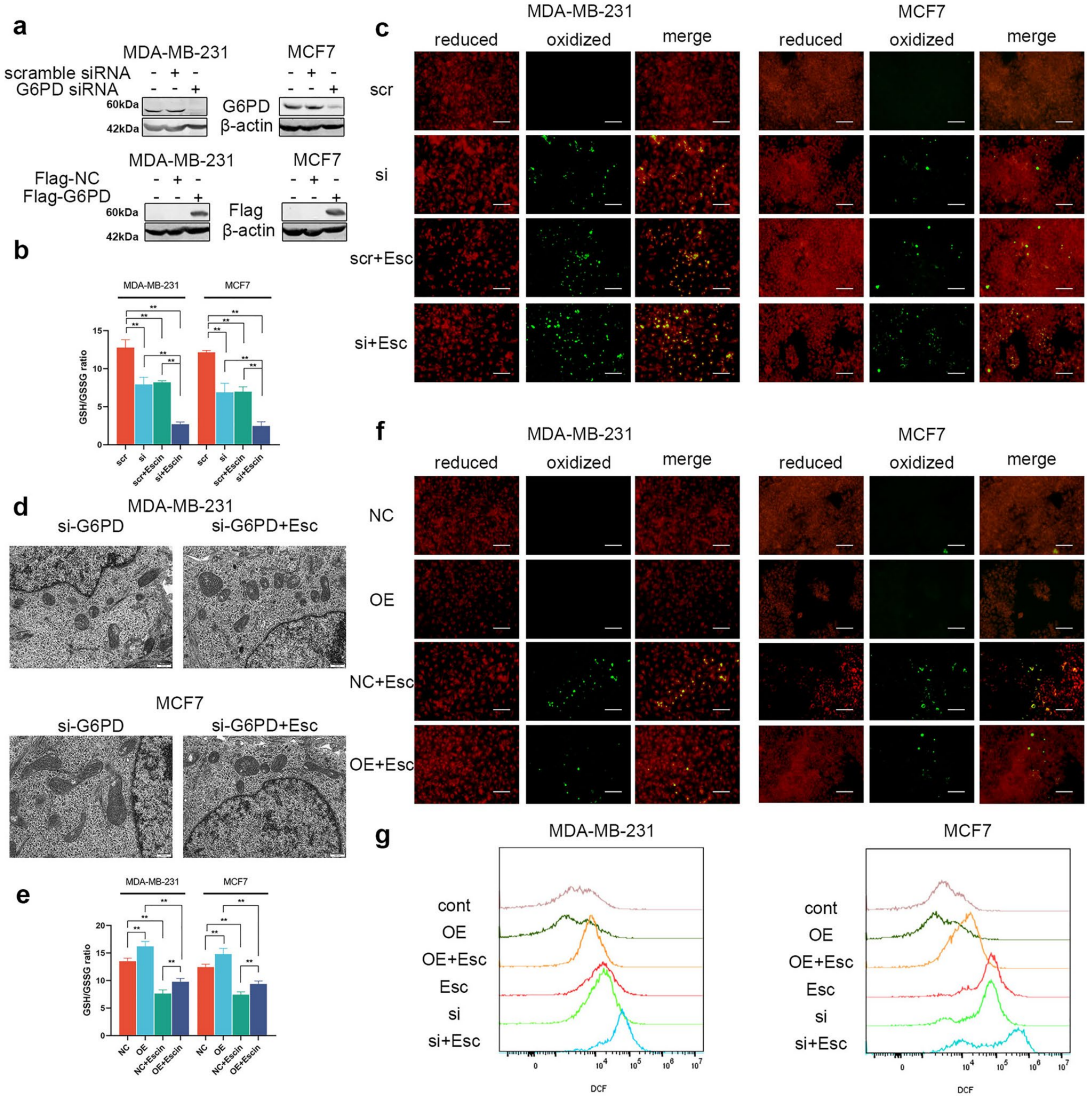
G6PD deficiency contributed to Escin-induced ferroptosis. **a** Knockdown and overexpression efficiency of G6PD in BC cells. **b**, **c** GSH/GSSG ratio and lipid peroxidation (200×, scale = 100 μm) measurement after G6PD knockdown with Escin treatment. **d** G6PD^KD^ cells were treated with or without Escin. Mitochondria were observed by electron microscopy (8000×, scale = 500 nm). **e**, **f** GSH/GSSG ratio and lipid peroxidation (200×, scale = 100 μm) measurement after G6PD overexpression with Escin treatment. **g** ROS levels were detected by flow cytometry

Next, the role of G6PD in Escin-induced ferroptosis was further verified after knockdown (KD) and overexpression (OE) of G6PD. The transfection efficiency is shown in Fig. 5a. G6PD deficiency depressed the GSH/GSSG ratio, and Escin exacerbated the depression (Fig. 5b). The level of lipid peroxidation demonstrated the same trends (Fig. 5c). Morphologically, knockdown of G6PD in BC cells led to mitochondrial impairment similar to that of Escin. In particular, the damage became more common in Escin-treated G6PD-deficient cells (Fig. 5d). Overexpressed cells demonstrated effects opposite those in G6PD^KD^ cells with regard to the GSH/GSSG ratio and lipid peroxidation (Fig. 5e, f). Furthermore, ROS levels in G6PD-overexpressing cells were slightly lower than those in control cells, while the ROS levels in G6PD-knockdown cells were enhanced. Escin treatment significantly increased intracellular ROS levels; this effect was relieved by G6PD overexpression and exacerbated by G6PD deficiency (Fig. 5g). In summary, G6PD inhibition favored Escin-induced ferroptosis.

### Escin induced ferroptosis through G6PD/GPX4 axis

**Fig. 6:**
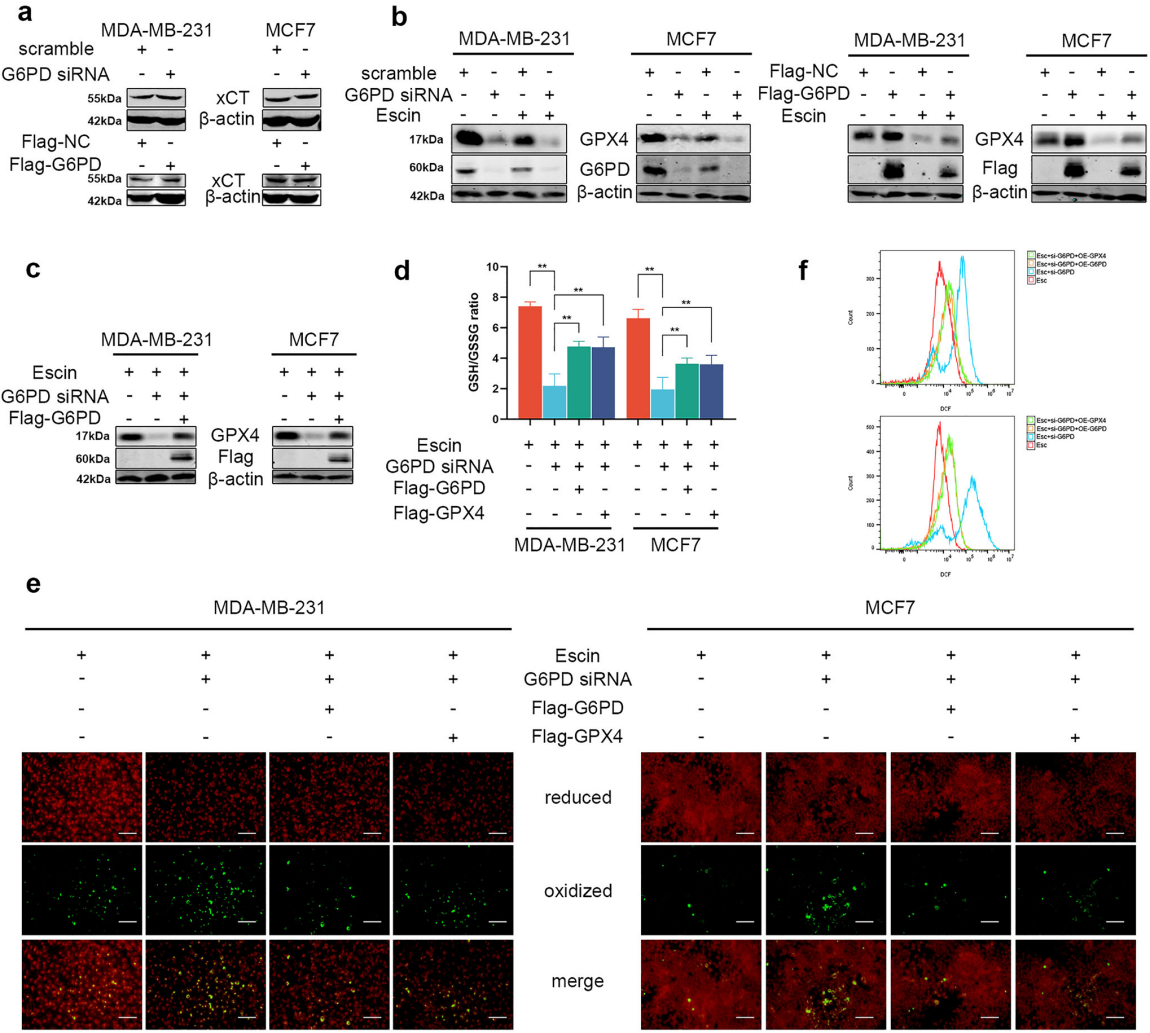
The G6PD/GPX4 axis was involved in Escin-induced ferroptosis. **a** The protein levels of xCT in G6PD^KD^ and G6PD^OE^ cells. **b** Cells were transiently transfected with G6PD siRNA or Flag-G6PD and their vector and then treated with 40 μg/ml Escin for 12 h. The protein level of GPX4 was detected. **c** After exposure to Escin, G6PD was overexpressed in G6PD^KD^ cells. The protein level of GPX4 was detected. **d**–**f** Cells were treated as described above. The GSH/GSSG ratio, lipid peroxidation (200×, scale = 100 μm) and ROS levels were measured

It is well-known that GPX4 was regulated by the level of GSH [9], which could be regulated by G6PD [35]. We subsequently try to verify whether GPX4 was mediated by G6PD. So, the expression of xCT and GPX4 was detected in G6PD-knockdown and G6PD-overexpressing cells. Consistently, the protein level of xCT was not significantly altered (Fig. 6a) when GPX4 was inhibited by G6PD knockdown and was further decreased in Escin-treated G6PD-knockdown cells (Fig. 6b). Similarly, GPX4 overexpression elevated the expression of GPX4 and reversed the inhibition of GPX4 by Escin (Fig. 6b). These results suggested that the activity of GPX4 was mediated by G6PD in Escin-induced ferroptosis.

Thus, we next investigated whether the G6PD/GPX4 axis was involved in Escin-induced ferroptosis. Overexpression of G6PD in G6PD^KD^ cells reversed the inhibition of GPX4 after Escin treatment (Fig. 6c). Furthermore, either G6PD or GPX4 overexpression reversed ferroptosis in G6PD^KD^ cells exposed to Escin (Fig. 6d–f). Overall, the G6PD/GPX4 axis was repressed in Escin-induced ferroptosis.

### Downregulation of G6PD exacerbated the antitumor effect of Escin in vivo

**Fig. 7:**
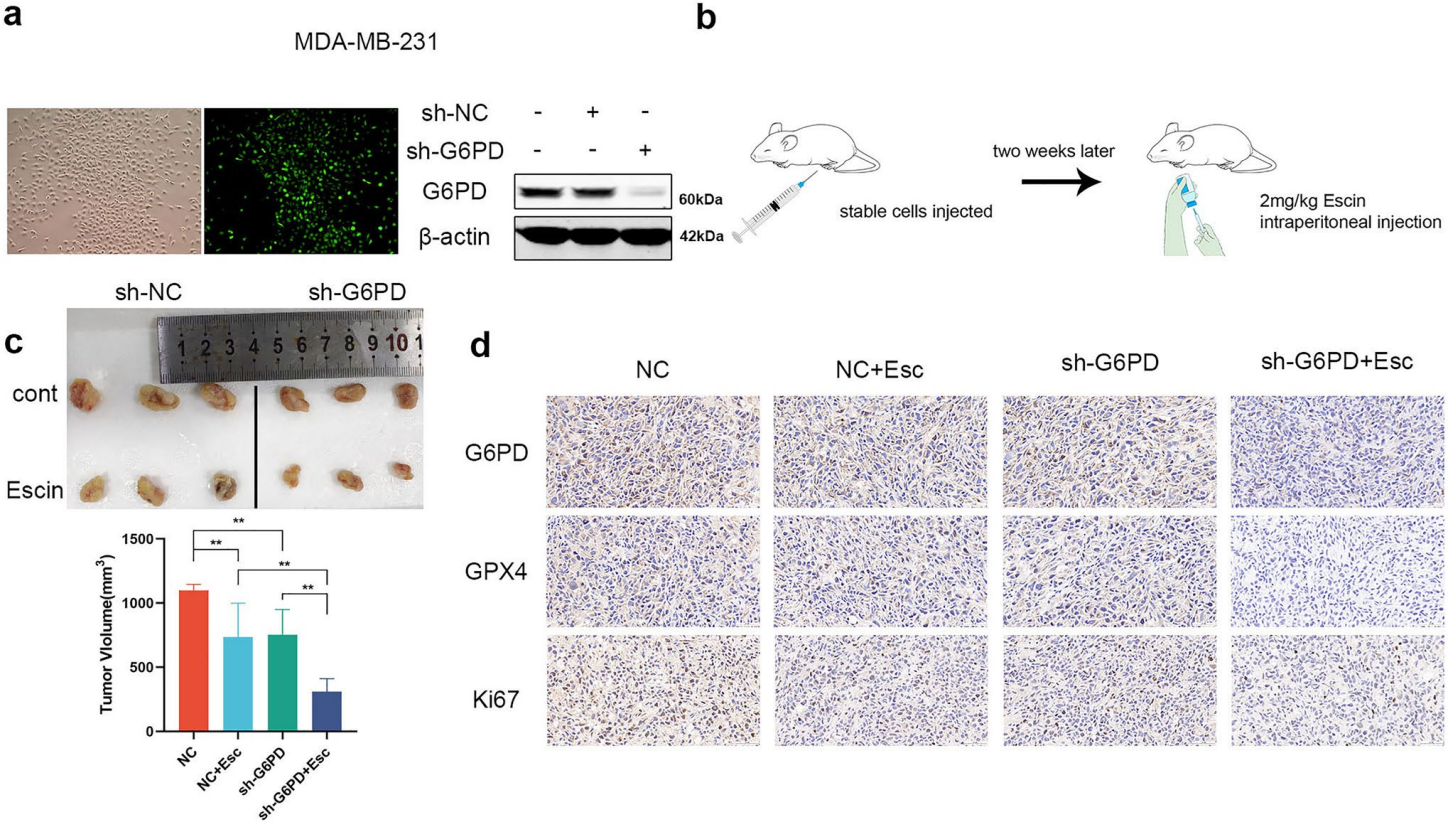
G6PD knockdown exacerbated the inhibition of tumor growth by Escin in vivo. **a** Transfection efficiency of sh-G6PD in MDA-MB-231 cells. **b** Procedure of the model establishment. **c** Comparison of tumor weight in various groups (n = 3). **d** IHC staining of xenografts

To evaluate the function of G6PD in the antitumor effect of Escin in vivo, xenografts of MDA-MB-231 cells transfected with vector or shRNA were examined. MDA-MB-231 cells stably expressing sh-NC and sh-G6PD were successfully established (Fig. 7a). 4-week-old female BALB/c nude mice were randomly divided into two groups and corresponding cells were injected into the right iliac fossa. When the tumor volume grew to approximately 60 mm^3^, half of these mice were randomly selected to be intraperitoneally injected with 2 mg/kg Escin, and the other half were intraperitoneally injected with an equal volume of normal saline as the control (Fig. 7b). The xenografts were removed and weighed when the volume reached 300 mm^3^. As shown in Fig. 7c, compared with the sh-NC group, both the Escin treatment group and the G6PD knockdown group showed slight inhibition of tumor growth. Furthermore, G6PD knockdown with Escin treatment achieved a more pronounced inhibition of tumor volume. The expression of G6PD, GPX4 and Ki67 in xenografts also confirmed the antitumor effect (Fig. 7d). In summary, inhibition of G6PD contributed to the antitumor effect of Escin in vivo.

### Escin synergized with DDP to promote cell death

**Fig. 8:**
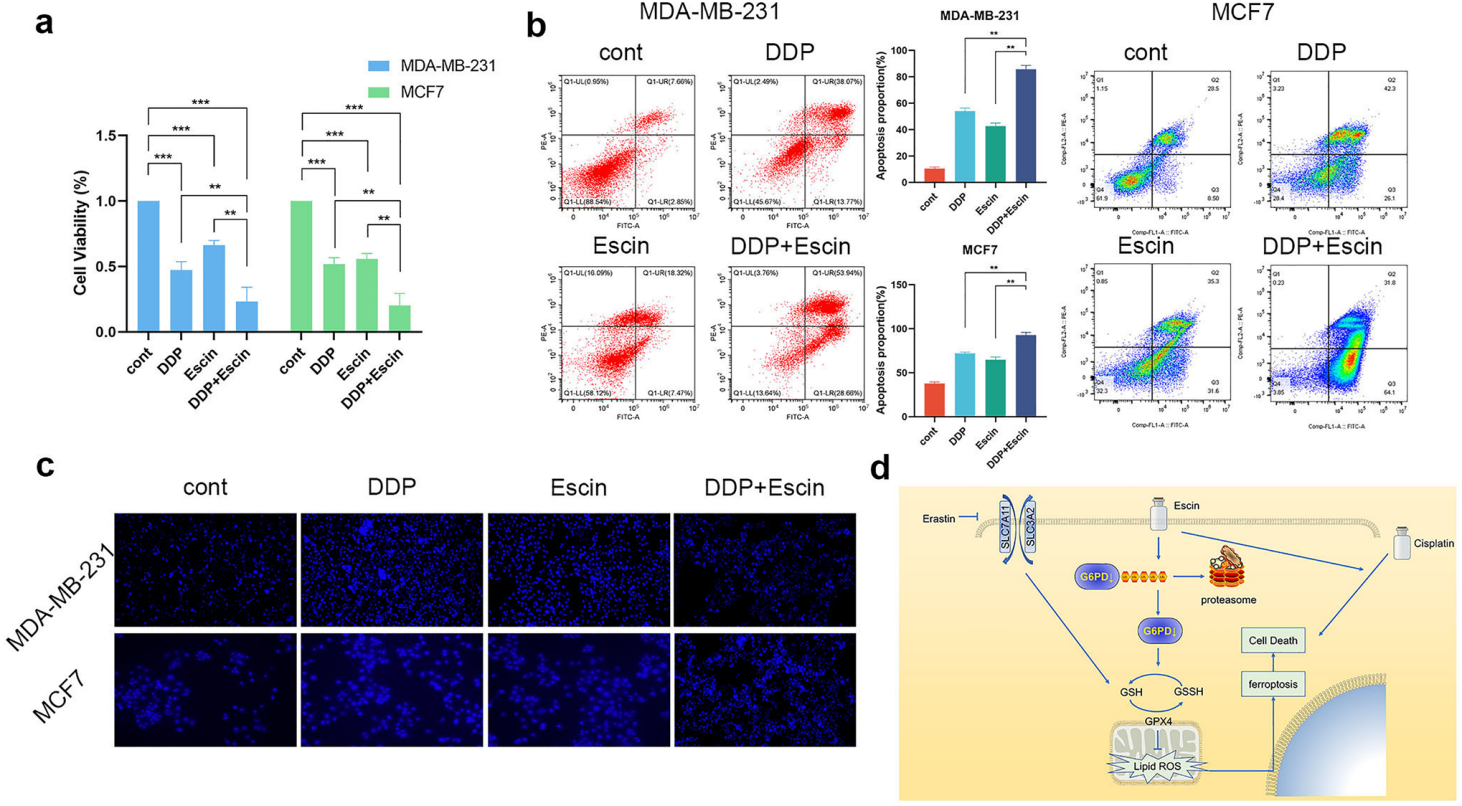
Escin synergized with DDP to induce apoptosis. **a** Cells were treated with DDP (1 μg/ml, 24 h), Escin (40 μg/ml, 12 h), or DDP + Escin. Cell viability was measured by CCK8 assay. **b** Cells were treated as described above. Apoptosis was analyzed by flow cytometry. **c** Hoechst staining in BC cells with the treatments described above. **d** Proposed model of Escin-triggered ferroptosis in BC cells

Given the potential application of ferroptosis inducers that synergize with traditional chemotherapeutic drugs, we examined whether Escin exacerbated the antitumor effect of DDP. Cell viability was measured after the corresponding treatments. Both DDP and Escin promoted BC cell death, while the combination of DDP and Escin resulted in more apoptosis (Fig. 8a–c). In short, Escin synergized with DDP to promote apoptosis.

## Discussion

Novel treatments might help many women who are threatened by BC. In this study, we found that Escin inhibited BC cell growth in vitro and in vivo. In vitro studies revealed that Escin reduced the GSH/GSSG ratio and elevated lipid peroxidation as well as ROS levels. Thus, Escin induced BC cell ferroptosis. Moreover, Escin facilitated G6PD ubiquitination, which led to the downregulation of GPX4. Knockdown of G6PD exacerbated Escin-induced ferroptosis, while overexpression of G6PD or GPX4 relieved this effect. An in vivo study also suggested that G6PD deficiency favored Escin-mediated inhibition of tumor growth. Finally, Escin synergized with DDP to trigger cell death, which suggests potential clinical applications (Fig. 8d).

Ferroptosis is characterized by iron-dependent lipid peroxidation. As the vital antioxidant defense system, system χ_c_^−^ transports cystine to sustain synthesis of GSH, the cofactor of GPX4 in reducing lipid peroxides, thereby reducing the accumulation of lipid ROS and subsequent ferroptotic cell death [36]. In our study, Escin did not affect xCT but inhibited GSH regeneration, thus causing lipid peroxidation and consequently ferroptosis. Furthermore, Escin-treated cells had a higher LC3II/I ratio and more autophagosomes than control cells, implying an increased autophagy level. The promoting effect of Escin on autophagic flux was also verified in our previous study, which demonstrated that p62 expression was elevated by Escin [26]. Numerous studies have suggested that ferroptosis can be autophagy dependent. For instance, NCOA4-mediated ferritinophagy causes iron accumulation, and RAB7A-mediated lipophagy, p62-mediated clockophagy and chaperone-mediated autophagy cause lipid peroxidation [37]. Thus, Escin serves as a ferroptosis inducer.

G6PD serves as not only a hub gene of ferroptosis but also an independent prognostic factor for BC patients [38]. In our study, electron microscopy images showed that G6PD deficiency led to a reduction in mitochondrial cristae and shrinkage of mitochondrial volume. The impairment of mitochondria may have arisen from uncontrolled intracellular redox homeostasis upon G6PD knockdown. G6PD is associated with the cytochrome P450 metabolic pathway. Cytochrome P450 oxidoreductase (POR) is upregulated in G6PD^KD^ HCC cells, which increases the peroxidation of membrane polyunsaturated phospholipids [39]. In addition, it is possible that Escin induced ferroptosis via G6PD inhibition. In our study, G6PD activity was decreased in Escin-treated BC. However, our previous study has revealed that p62 is upregulated by Escin in colorectal cancer [26]. In addition, another study in gastric cancer has illustrated that p62 contributes to the translocation of Nrf2 into the nucleus [40]. G6PD has been verified to be upregulated by Nrf2, which leads to metabolic reprogramming and alleviates ferroptosis in sepsis-induced vascular leakage [41]. The difference between our results and those of the previous studies could be explained by the different cell lines and disease models and, more importantly, the sensitivity to Escin. During our experiments, the expression of Nrf2 in the nucleus and cytoplasm was not significantly changed after Escin exposure (data not shown). It is likely that the Nrf2 pathway does not respond to Escin treatment in BC.

Numerous studies have reported such contradictions regarding Escin-induced increases in ROS levels during Escin-triggered cell death. Although the antitumor effect of Escin has been confirmed [24, 26, 42], the role of the elevation in ROS level induced by Escin has remained elusive. In our study, Escin inhibited the G6PD/GPX4 axis, thereby increasing lipid peroxidation and ROS levels and leading to ferroptosis. The findings of a study in human renal cancer and osteosarcoma also support our viewpoint, revealing that Escin induces apoptosis via ROS generation and that antioxidants such as NAC can alleviate its cytotoxicity [42, 43]. However, ROS inhibitors contribute to Escin-induced apoptosis in HCC and colon carcinoma [27]. It is likely that the levels of ROS, as protective factors, are enhanced in response to Escin treatment. Simultaneously, ROS are produced as byproducts of Escin-triggered ferroptosis. The different advantageous pathways in various cancer cells result in contrary effects in response to the same reagent.

In terms of clinical treatments, the synergistic effects of Escin and 5-FU have been verified in MCF7 cells. The combination of these agents increases apoptosis via p53 upregulation and bcl-2 downregulation [28]. Similarly, our study findings implied that Escin synergized with DDP to augment cell death in MDA-MB-231 and MCF7 cells. Additionally, the χ_c_^−^/GSH/GPX4 axis contributes to the regulation of tumor chemoresistance. For example, cells with increased GSH levels exhibited increased resistance to chemical drugs, such as doxorubicin [44], DDP [45], and 5-FU [46]. Accordingly, although the synergistic effect of Escin and DDP needs further exploration in drug-resistant cell lines, Escin may attenuate the drug resistance caused by increased GSH levels.

Some limitations necessitate deeper exploration. First, although we illustrated that an autophagy inhibitor rescued G6PD activity, the degradation process requires more evidence. Additionally, whether other molecules are involved in Escin-modulated G6PD expression should be determined. Second, the differences among various disease models in the response to Escin deserve more investigation. Sequencing analysis could be helpful in elucidating the known pathways that are activated in response to Escin in different cancer cells.

In conclusion, our study reveals that Escin facilitates the ubiquitination and degradation of G6PD. The resulting G6PD deficiency contributes to GPX4 downregulation and ferroptosis. Finally, Escin synergizes with DDP to induce apoptosis in BC.

## Data Availability

The datasets generated during and/or analysed during the current study are available from the corresponding author on reasonable request.
